# Recombination analysis reveals a double recombination event in hepatitis E virus

**DOI:** 10.1186/1743-422X-7-129

**Published:** 2010-06-14

**Authors:** Hua Wang, Wen Zhang, Bin Ni, Hongxing Shen, Yuyu Song, Xiaochun Wang, Shihe Shao, Xiuguo Hua, Li Cui

**Affiliations:** 1 School of Medical Science and Laboratory Medicine, Jiangsu University, 301 Xuefu Road, Zhenjiang, Jiangsu 212013, China; 2 School of Agriculture and Biology, Shanghai JiaoTong University, 800 Dongchuan Road, Shanghai 200240, China

## Abstract

Recombination of Hepatitis E Virus (HEV) has rarely been reported. In the present study, phylogenetic and recombination analyses were performed on 134 complete HEV genomes. Three potentially significant recombination events, including both intra-genotype and one inter-genotype, were identified by recombination detection analysis. Recombination events I and II occurred intra-genotype and inter-genotype, respectively, among three isolates, including the lineage represented by CHN-XJ-SW13 (GU119961, swine isolate), E067-SIJ05C (AB369690, human isolate), and JJT-Kan (AB091394, human isolate), and lead to the recombinant swine isolate swCH31 (DQ450072). Recombination event III occurred between the lineage represented by the NA1 (M73218) and K52-87 (L25595), which resulted in the recombinant Xingjiang-1 (D11092). Our analyses proved that that recombination could occur between human and swine HEV strains, double recombination events existed in HEV, and recombination event could happen within ORF2 region of HEV. These results will provide valuable hints for future research on HEV diversity.

## Findings

Hepatitis E virus (HEV), a member of the genus Hepevirus, is a non-enveloped virus with a positives-stranded RNA genome of approximately 7.2 kb in length (Reyes et al., 1990). HEV is believed to be transmitted by the faecal-oral route and its infection affects primarily young adults and is generally mild [1,2]. The mortality rate of HEV infection is higher among women, and hepatitis E virus infection is highly prevalent among pregnant women [3,4]. HEV and antibodies to HEV have been reportedly found in a wide variety of animals, especially swine [5-8]. A hypothesis has arisen that zoonosis is involved in the transmission of HEV.

The HEV genome has three partially overlapped open reading frames (ORFs). ORF1 is located at the 5'-terminus of the genome and encodes non-structural proteins. ORF2 is at the 3'-terminus of the HEV genome and encodes the viral capsid protein which has three glycosylation sites. ORF3 overlaps with either ORF1 or ORF2 [9]. HEV isolates were divided into four distinct genotypes according to sequence and phylogenetic analyses. Genotype 1 was previously believed to only infect humans, but reportedly detected from a pig in Cambodia recently [10]. Genotype 2 has only been identified in humans in Mexico and Africa (Nigeria, Chad). Genotype 3 is prevalent in swine herds and humans all over the world. Genotype 4 HEV is mainly distributed in China, Japan, India, Indonesia, and Vietnam. Genotype 4 HEV has a wide host range, being prevalent in humans, swine, and some other animals. These four types of virus are thought to comprise a single serotype [9].

Recombination is a relatively common phenomenon in positive-sense RNA viruses [11-13] and understanding recombination can be helpful in unravelling the evolution of pathogens and drug resistance. So far, two reports revealed the presence of HEV recombination. However, one of them was performed in 2005, when there were only about 30 HEV strains with full genome available in GenBank [14]; the other one was focused on the open reading frame structure analysis [15]. In the present study, therefore, we analyze the available complete HEV genome sequences in GenBank in order to systematically investigate the presence of recombination among HEV strains.

**Figure 1:**
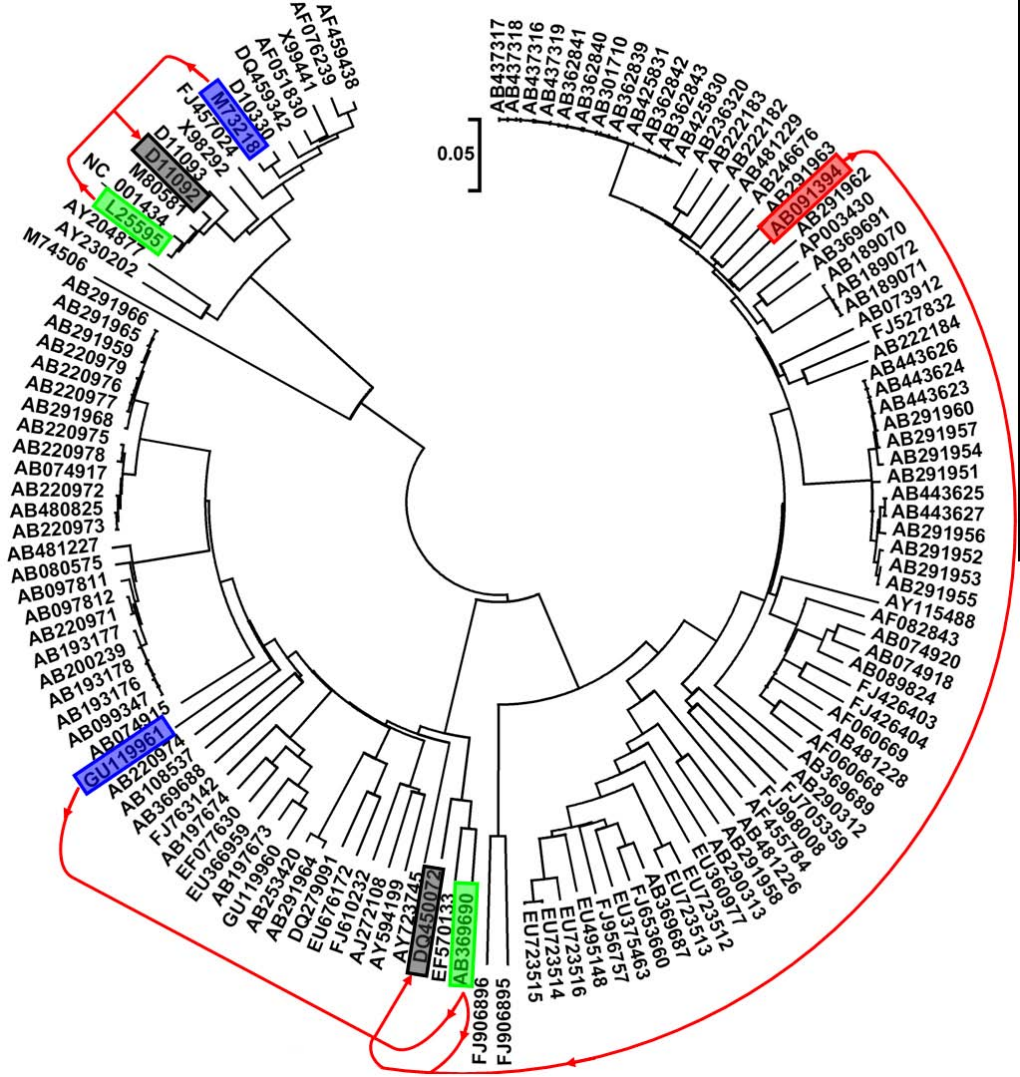
**Phylogenetic tree for the 134 HEV genomes**. The black shadow boxes indicated the two recombinants: DQ450072 and D11092. The green, blue, and red shadow boxes showed the parental isolates. The red line presented the recombination event.

The study sequences comprised all of the available complete genome sequences of HEV from GenBank dated November 2009. Sequences were firstly screened to exclude patented and artificial mutants, and then aligned in the ClustalW program. The alignment was manually adjusted for the correct reading frame. The 5'-terminus non-coding region (about 25 nt) is highly conserved within all known 4 genotypes, therefore this region were removed before re-alignment. The remaining 134 HEV genomes were re-aligned to generate a phylogenetic tree using the neighbor-joining (NJ) method in MEGA4 [16] with the Kimura 2-parameter model (Fig 1). The genotypes of these HEV genomes based on the phylogenetic tree were consistent with the genotype information from the original sources. Detection of potential recombinant sequences, identification of potential parental sequences, and localization of possible recombination break points were determined using the Recombination Detection Program (RDP) [17], GENECONV [18], BOOTSCAN [19], MaxChi [20], CHIMAERA [21], and SISCAN [22] methods embedded in RDP3 [23]. A Multiple-comparison-corrected P-value cutoff of 0.05 was used throughout.

**Figure 2:**
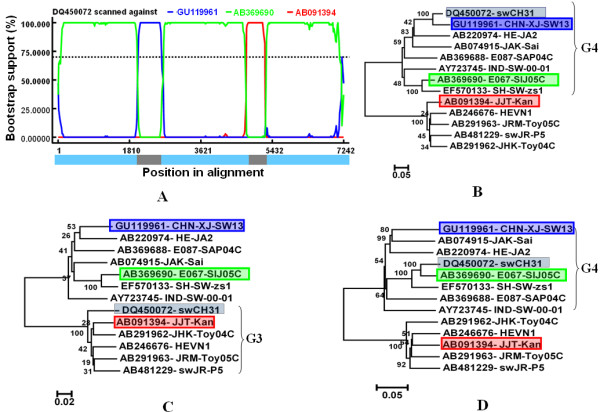
**Identification of recombination event I and II**. (A) BOOTSCAN evidence for the recombination origin on the basis of pairwise distance, modeled with a window size 200, step size 20, and 100 Bootstrap replicates; (B) Neighbor joining tree (2,000 replicates, Kimura 2-parameter distance) constructed using the recombinant region (2021-2618 nt); (C) Neighbor joining tree constructed using the recombinant region (4754-5201 nt); (D) Neighbor joining tree constructed using the non-recombinant regions consisted of the rest of the genome. The green, blue, and red shadow boxes showed the parental isolates and the black boxes showed the daughter isolate.

Three potentially significant recombination events were found with a high degree of confidence judged by the above-mentioned six recombination detection methods (Table 1). Fig 2A indicated the BOOTSCAN plot of the double recombination events (event I and event II) which lead to an interesting recombinant swCH31 (DQ450072). Event I belonged to intra-genotype recombination and happened between the lineage represented by the strain CHN-XJ-SW13 (GU119961) as the major parent and the strain E067-SIJ05C (AB369690) lineage as the minor parent. CHN-XJ-SW13 is a Chinese genotype 4 strain which was isolated from pigs in Xinjiang province of China, while E067-SIJ05C is a Japanese genotype 4 strain which was isolated from a Japanese patient who had traveled to Shanghai of China before hepatitis E onset. Event II belonged to inter-genotype recombination, which occurred between strain E067-SIJ05C as the major parent and a genotype 3 strain JJT-Kan (AB091394) as the minor parent that was isolated from a patient with acute hepatitis in Japan. To confirm the results, the relevant strains were analyzed by neighbor joining trees using MEGA4 (Fig 2B, C, and 2D). Fig. 2B, C, and 2D indicated the trees constructed on the recombinant region in event I, event II, and the non-recombinant regions, respectively. The recombinant strain swCH31clustered closely with its three parental strains in the three phylogenetic trees, respectively. The results clearly indicate the existence of the double recombination events between CHN-XJ-SW13, E067-SIJ05C, and JJT-Kan. A previous report indicated that DQ450072 not only had a mosaic ORF structure but was putative inter-genotype recombinant [15]. The present study revealed that this recombinant swCH31 was produced by both inter- and intra genotype recombination which occurred among three potential parental strains belonging to two different genotypes.

**Table 1:** The average P-value of three recombinant events analyzed by six recombination detection methods

Event	RDP	GENOCOV	BOOTSCAN	MaxChi	CHIMAERA	SISCAN
I	3.59 × 10^-12^	5.56 × 10^-24^	1.31 × 10^-39^	8.31 × 10^-18^	7.55 × 10^-10^	1.52 × 10^-17^
II	4.18 × 10^-19^	1.25 × 10^-25^	1.26 × 10^-45^	1.67 × 10^-16^	3.77 × 10^-18^	5.69 × 10^-16^
III	6.73 × 10^-10^	1.78 × 10^-6^	6.57 × 10^-10^	5.76 × 10^-6^	4.31 × 10^-5^	1.47 × 10^-4^

Recombination within the capsid gene has been suggested for other positive-strand RNA virus such as norovirus [24,25]. The recombination of the virus capsid gene may play a key role in virulence, allowing new recombinants to evade immune response and possible viral extinction. ORF2 of HEV encodes the capsid protein, which contains the antigenic regions and partial nucleotide sequence of ORF2 is predicted to be well suited for phylogenetic classification of HEV [26,27]. In the present study, we detected that recombination event II occurred within the capsid gene of HEV (Fig 2A), and such mutations in the capsid gene will produce a protein which is better able to evade the host immune response, thereby allowing higher viral titer and greater overall fitness. Moreover, we should notice that recombination event II happened between human (E067-SIJ05C) and swine (CHN-XJ-SW13) HEV isolates. This suggested that recombination of HEV can occurred between viruses infecting different host species, which needs to be recorded, as they have serious implications for the future evolution of infectious agents.

**Figure 3:**
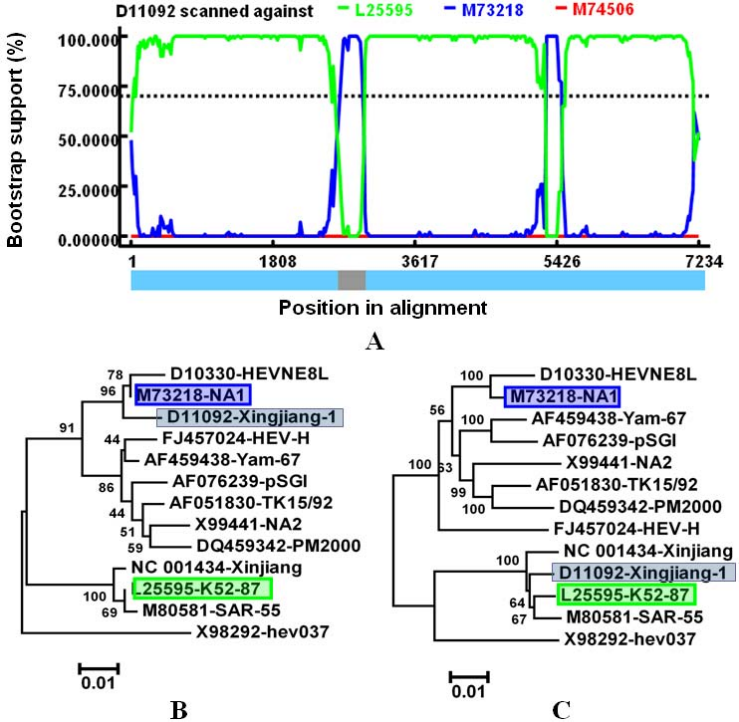
**Identification of recombination event III**. (A)BOOTSCAN evidence for the recombination origin on the basis of pairwise distance, modeled with a window size 200, step size 20, and 100 Bootstrap replicates; (B)Neighbor joining tree constructed using the recombinant region (2592-2941nt); (C)Neighbor joining tree constructed using the non-recombinant regions consisted of the rest of the genome. The green and red shadow boxes showed the parental isolates and the black shadow boxes showed the daughter isolate.

Our analysis suggested that Xingjiang-1 (D11092) was a potential recombinant between lineage NA1 (M73218) as the minor parent and K52-87 (L25595) as the major parent (Fig 3A). Xingjiang-1 belonged to genotype 1 and was isolated from the Xinjiang epidemic (1986-1988) of China [28]. The two parental strains, NA1 and K52-8, were also genotype 1 strains and isolated from Xinjiang of China in different labs [29,30], suggesting that Xingjiang-1 may be originated from recombination events during the HE outbreak in Xinjiang of China from 1986-1988. This recombination event was also confirmed by the phylogenetic analysis based on the relevant strains (Fig 3B and 3C). From the phylogenetic trees, we can see that the recombinant clustered closely with its minor parental strain NA1 and major parental strain K52-87 in Fig 3B and 3C, respectively.

In our study, Uigh179 (D11093) was revealed to be a potential recombinant (Additional file 1: Fig.S1), which is consistent with a previous report [14]. However, the potential parental strains of this recombinant were different between the present study and previous study. Van's study didn't include the strain Xingjiang-1(D11092) which was indicated to be one of the most probable parental strains in the present study. The strain information of Uigh179 and Xingjiang-1 showed that the two strains were sequenced in the same lab in Nihon University School of Medicine in 1992. Therefore, it should be cared whether this recombination event non-naturally occurred by sequencing error and/or contamination in the lab. A genotype 3 HEV strains swJ13-1 (AB097811) was also found to be a potential recombinant in the present study (Additional file 2: Fig S2). It was isolated from a 4-month-old pig in 2002 in Japan [31]. Our study suggested this recombination event occurred between HE-JA1 (AB097812) and swJB-H7 (AB481227). The potential recombinant swJ13-1 and its parental strain HE-JA1, which was isolated from a Japanese patient, were determined in the same lab and shared 99.0% sequence identity over the complete genome. The ORF2 and ORF3 of the two strains were even identical to each other [31]. It is therefore tempting to speculate that this recombination event might happen non-naturally in the lab.

Taken together, we analyzed 134 non-redundant HEV complete genomes using detailed phylogenetic and recombination analytic methods and identified two recombinants (swCH31 and Xingjiang-1), and swCH31 was proved to be produced by double recombination events which occurred among three potential parental strains belonging to two different genotypes. Moreover, it should be noted that recombination could occur between human and swine HEV strains, double recombination events existed in HEV, and recombination event can happen within ORF2 region of HEV. Other two isolates (AB097811 and D11093) may be potential non-natural recombinants happened in the lab. The present study could reminder us that recombination also contribute to the genetic variety of HEV.

## Competing interests

The authors declare that they have no competing interests.

## Authors' contributions

WZ and LC conceived the study. All authors performed recombination analysis and critically reviewed and approved the final manuscript. WZ wrote the paper.

## Supplementary Material

Additional file 1 **Identification of recombinant Uigh179**. (A) BOOTSCAN evidence for the recombination origin on the basis of pairwise distance, modeled with a window size 200, step size 20, and 100 Bootstrap replicates; (B) Neighbor joining tree constructed using the recombinant region (1018-1979nt); (C) Neighbor joining tree constructed using the non-recombinant regions consisted of the rest of the genome. Click here for file

Additional file 2 **Identification of recombinant swJB-H7**. (A) BOOTSCAN evidence for the recombination origin on the basis of pairwise distance, modeled with a window size 200, step size 20, and 100 Bootstrap replicates; (B) Neighbor joining tree constructed using the recombinant region (1177-2805nt); (C) Neighbor joining tree constructed using the non-recombinant regions consisted of the rest of the genome. Click here for file
