# Supplementary figures and images for: Recombination analysis reveals a double recombination event in hepatitis E virus
Source: Virol J. 2010 Jun 14;7:129. doi: 10.1186/1743-422X-7-129 (PMC2901267; doi:10.1186/1743-422X-7-129)

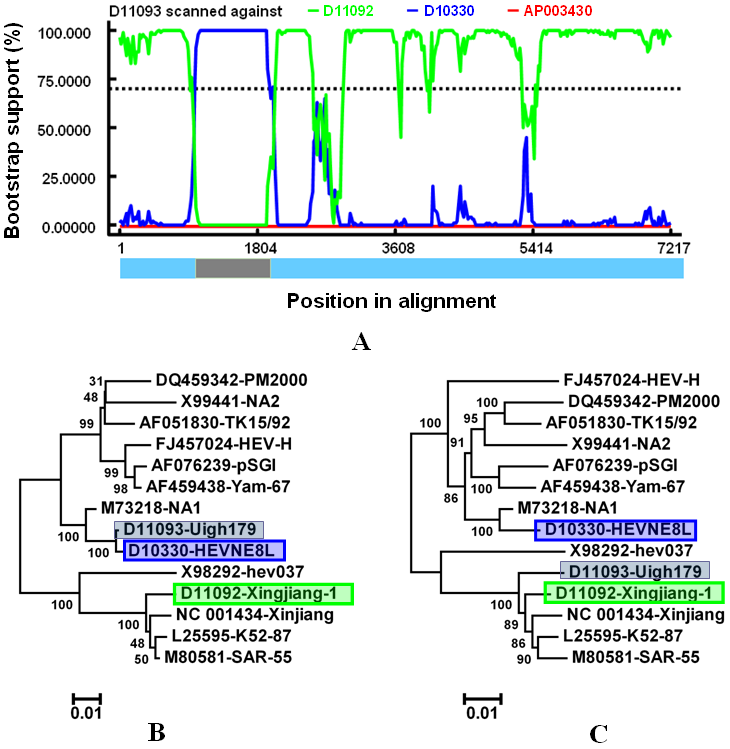

Supplement: Additional file 1 — Identification of recombinant Uigh179. (A) BOOTSCAN evidence for the recombination origin on the basis of pairwise distance, modeled with a window size 200, step size 20, and 100 Bootstrap replicates; (B) Neighbor joining tree constructed using the recombinant region (1018-1979nt); (C) Neighbor joining tree constructed using the non-recombinant regions consisted of the rest of the genome. [file 1743-422X-7-129-S1.TIFF]

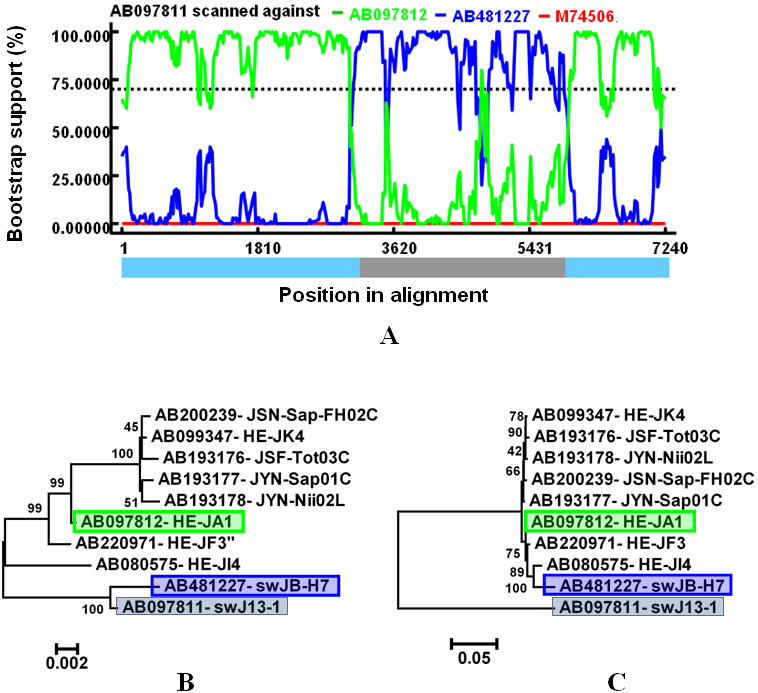

Supplement: Additional file 2 — Identification of recombinant swJB-H7. (A) BOOTSCAN evidence for the recombination origin on the basis of pairwise distance, modeled with a window size 200, step size 20, and 100 Bootstrap replicates; (B) Neighbor joining tree constructed using the recombinant region (1177-2805nt); (C) Neighbor joining tree constructed using the non-recombinant regions consisted of the rest of the genome. [file 1743-422X-7-129-S2.TIFF]
